# Cohort profile update: The Cork and Kerry Diabetes and Heart Disease Study

**DOI:** 10.12688/hrbopenres.13626.1

**Published:** 2023-04-21

**Authors:** Danko Stamenic, Janas M. Harrington, Seán R. Millar, Lisa Delaney, Katarzyna A. Gajewska, Claire M. Buckley, Sheena M. McHugh, Ivan J. Perry, Patricia M. Kearney

**Affiliations:** 1 School of Public Health, University College Cork, Cork, Co. Cork, Ireland

**Keywords:** Rescreen, Cohort, Diabetes, Hypertension, Cardiovascular Disease

## Abstract

**Background: **The Cork and Kerry Diabetes and Heart Disease Study was established to investigate the prevalence of diabetes and cardiovascular disease among middle-aged adults in Ireland. The Mitchelstown cohort was recruited from a single large primary care centre between 2010–2011. A rescreen of this cohort was conducted in 2015.

**Methods: **Data were collected on cardiovascular health and associated risk factors. In addition, the rescreen incorporated new measures which included information on cognition and frailty, medication adherence, dietary factors and the collection of stool samples with RNA sequencing of the gut microbiome.

**Results: **Of 2047 participants in the original cohort, 237 (11.6%) were deceased, too ill to participate or were lost to follow-up. Of the remaining 1810 baseline study participants, 1378 men and women aged 51–77 years agreed to take part in the rescreen (response rate of 76.1%). The prevalence of hypertension was high, ranging from 50% to 64% depending on the measurement method. An investigation of the association of gut microbiota with metabolic syndrome and obesity indicated greater microbiome diversity in metabolically healthy non-obese individuals relative to their unhealthy counterparts. Analysis of prescribing data over time demonstrated a high prevalence of potentially inappropriate prescribing among older-aged people in primary care which increased as they progressed to more advanced old age.

**Conclusions: **The rescreen has provided new insights into cardiovascular health. In addition, this study is embedded in a single primary care centre, enabling passive follow-up of study participants through electronic health records. All data collected at baseline and rescreen are maintained and stored at the School of Public Health, University College Cork and specific proposals for future collaborations are welcome.

## The original cohort

**Figure 1.**
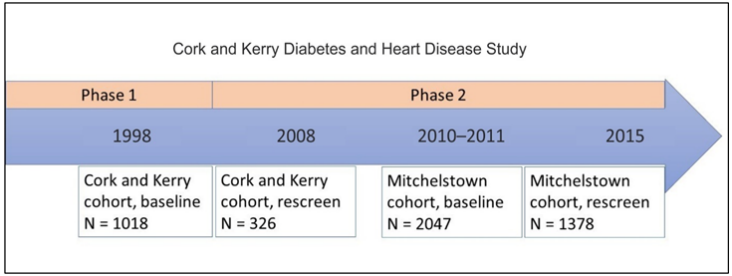
Timeframe of the Cork and Kerry Diabetes and Heart Disease Study.

The Cork and Kerry Diabetes and Heart Disease Study comprises two separate cohorts: the Cork and Kerry cohort and the Mitchelstown cohort ^1, 2^. The two cohorts were recruited from a similar region in the south of Ireland among a similar age population and both focused on cardiovascular health. However, the cohorts were begun at different timepoints and utilised a different sampling approach ( Figure 1). The Cork and Kerry cohort comprised patients from 17 individual general practices in counties Cork and Kerry, who were recruited in 1998, and underwent follow-up assessment in 2008 ^2^. The Mitchelstown cohort was recruited from a single large primary care centre: the Livinghealth Clinic in Mitchelstown, County Cork, between 2010 and 2011.

The Cork and Kerry cohort was established to provide a profile of the glucose tolerance status, cardiovascular health and related factors in an Irish adult general population sample. Follow-up of the cohort and recruitment of a new cohort (the Mitchelstown cohort) provided an opportunity to compare the prevalence of cardiovascular risk factors in the population of the region over time and to further evaluate the prevalence of ideal cardiovascular health ^3^. In addition, the Mitchelstown cohort incorporated novel measures of cardiovascular risk such as blood pressure variability and subclinical vascular disease ^4^.

## Why was there a need for a significant change in data collected?

There are two main reasons for the cohort profile update. First, additional data are available since the cohort profile was published. Stored samples from the first wave of the Mitchelstown cohort were analysed for inflammatory biomarker profiling and lipoprotein particle subclass size and concentrations determined using nuclear magnetic resonance spectroscopy. This has provided an opportunity for novel investigation of relationships between biomarkers and cardiovascular risk ^5– 9^. In addition, as the Mitchelstown cohort is embedded in a single large primary care centre, annual information on the cohort is available from electronic patient records; these data are provided in an ‘annual sweep’, with detailed information on prescriptions, new diagnoses and specialist referrals. In terms of survey data, between 2015 and 2017 a full rescreen of the Mitchelstown cohort was undertaken. This comprised of general health questionnaires (GHQ) and dietary assessment as well as physical assessments which included anthropometrics, ambulatory blood pressure monitoring and objective measurement of physical activity over one week using accelerometers. Blood, urine and stool samples were also collected. Second, new data were gathered in the rescreen in response to the development of new collaborations and additional capacity in key areas which included population ageing, medication use and the role of the microbiome with regard to cardiometabolic health. This paper provides an update on data collected and describes the refined research focus.

## What are the new areas of research and new data collection?

In addition to providing an update on the prevalence of diabetes, cardiovascular health and associated risk factors in the Mitchelstown cohort, new data were collected in the rescreen. The new areas of research include population ageing, with inclusion of measures of cognitive function, frailty, sarcopenia and assessment of gait speed and grip strength as well as medication use and medication adherence, with detailed information on antibiotic/anti-inflammatory drug use and medication prescribing. Sub-studies include the collection of stool samples with RNA sequencing of the gut microbiome to assess relationships between the microbiome and measures of cardiometabolic health, the validation of a shortened Food Frequency Questionnaire (FFQ) and objective measurement of physical activity using accelerometers, allowing detailed assessment of different types of activity. The availability of comprehensive dietary and physical activity data provides an opportunity to assess adherence to World Cancer Research Fund guidelines on diet and exercise ^10^. Population attributable risks will be estimated for several cancers based on the prevalence of adherence in this sample of older Irish adults. 

## Who is in the cohort?

Data collection for the rescreen commenced in 2015. Of the 2047 in the cohort, 237 participants (11.6%) were deceased, too ill to participate or were lost to follow-up. Of the remaining 1810 baseline study participants, 1378 agreed to take part in the rescreen (response rate of 76.1%). A comparison of baseline sociodemographic/general health characteristics and cardiovascular disease risk factors of the participants at baseline for the overall cohort, and for rescreen respondents, non-respondents and two of the sub-studies are provided in Table 1. Overall, those who participated in the rescreen were broadly like the baseline sample in terms of sociodemographic characteristics, self-rated health and cardiovascular risk factors including blood pressure, physical activity, current smoking status and overweight/obesity assessed using body mass index (BMI) categories ^11^. Non-respondents included a higher proportion of those with a primary education only. The microbiome sub-study was restricted to individuals aged 55 years and over at the time of rescreen. There was a higher proportion of males in the accelerometer study than those in the overall rescreen.

**Table 1.** Comparison of baseline characteristics of Mitchelstown cohort study participants.

Variable	Baseline Mitchelstown cohort N=2047	Completed Rescreen N=1378	Non- respondents N=432	Microbiome sub-study N=382	Accelerometer sub-study N=366
Age category (years)					
*Under 55*	512 (25.0%)	362 (26.3%)	90 (20.8%)	-	105 (28.7%)
*55–59*	557 (27.2%)	388 (28.2%)	108 (25.0%)	34 (8.9%)	110 (30.1%)
*60–64*	549 (26.8%)	379 (27.5%)	112 (25.9%)	221 (57.8%)	98 (26.8%)
*65–69*	393 (19.2%)	232 (16.8%)	110 (25.5%)	116 (30.4%)	50 (13.7%)
*70+*	36 (1.8%)	17 (1.2%)	12 (2.8%)	11 (2.9%)	3 (0.1%)
Gender					
*Male*	1008 (49.2%)	698 (50.7%)	182 (42.1%)	194 (50.8%)	199 (54.4%)
*Female*	1039 (51.8%)	680 (49.3%)	250 (57.9%)	188 (49.2%)	167 (45.6%)
Education					
*Primary only*	537 (23.6%)	318 (25.0%)	139 (34.1%)	118 (30.9%)	78 (23.0%)
*Secondary only*	936 (49.1%)	644 (50.5%)	192 (47.2%)	169 (44.2%)	177 (52.2%)
*Tertiary*	435 (22.8%)	312 (24.5%)	76 (18.7%)	67 (17.5%)	84 (24.8%)
Marital status					
*Single*	133 (6.6%)	108 (7.9%)	45 (10.9%)	24 (6.4%)	25 (6.9%)
*Married/co-habiting*	1586 (78.7%)	1099 (80.8%)	312 (75.5%)	305 (81.1%)	307 (84.8%)
*Divorced/separated*	177 (8.8%)	81 (6.0%)	34 (8.2%)	18 (4.8%)	20 (5.5%)
*Widowed*	119 (5.9%)	72 (5.3%)	32 (7.7%)	29 (7.7%)	10 (2.8%)
Self-rated health					
*Very good*	590 (29.5%)	407 (30.1%)	124 (29.5%)	95 (25.3%)	114 (31.8%)
*Good*	1098 (54.8%)	760 (56.2%)	216 (51.4%)	219 (58.4%)	203 (56.5%)
*Fair*	272 (13.6%)	161 (11.9%)	72 (17.1%)	54 (14.4%)	36 (10.0%)
*Poor*	34 (1.7%)	20 (1.5%)	7 (1.7%)	7 (1.9%)	5 (1.4%)
*Very poor*	9 (0.4%)	4 (0.3%)	1 (0.2%)	-	1 (0.3%)
Current smoker	292 (14.8%)	179 (13.5%)	66 (16.1%)	31 (8.4%)	49 (13.9%)
Physical activity (IPAQ)					
*High*	420 (21.9%)	290 (22.3%)	87 (21.7%)	82 (22.9%)	76 (22.0%)
*Moderate*	566 (29.5%)	393 (30.2%)	122 (30.4%)	111 (31.0%)	115 (33.2%)
*Low*	932 (48.6%)	616 (47.5%)	192 (47.9%)	165 (46.1%)	155 (44.8%)
Mean BMI (SD)	28.6 (4.8)	28.6 (4.6)	28.6 (5.0)	29.0 (4.6)	28.3 (4.4)
BMI category					
*Normal weight* *	447 (21.9%)	298 (21.7%)	86 (20.0%)	72 (18.9%)	85 (23.3%)
*Overweight*	925 (45.3%)	635 (46.3%)	202 (47.1%)	169 (44.4%)	166 (45.5%)
*Obese*	668 (32.8%)	443 (32.0%)	141 (32.9%)	140 (36.7%)	114 (31.2%)
Mean diastolic blood pressure (SD)	80.2 (9.8)	79.9 (9.5)	80.2 (10.3)	79.9 (9.4)	79.3 (9.5)
Mean systolic blood pressure (SD)	129.6 (16.9)	129.1 (16.1)	130.3 (18.8)	131.2 (16.5)	128.7 (16.0)

Numbers and % are shown. Continuous variables are shown as a mean and standard deviation (SD). *Includes 7 participants with BMI <18.5.

## What has been measured?

All participants who agreed to take part in the rescreen study were invited to attend the Livinghealth Clinic in Mitchelstown on two occasions. Before the first study visit, participants were asked to complete a GHQ and long-form FFQ. In addition to these self-completed questionnaires, all participants were asked to bring the first morning specimen of urine voided on the day of the appointment for estimation of urinary albumin/creatinine ratio, sodium and potassium. Participants in the microbiome sub-study provided a stool sample and completed a Barthel Index of Activities of Daily Living questionnaire ^12^, administered via telephone interview prior to their appointment. During the first study visit, fasting blood samples were taken and processed for assessment of several biochemistry and haematology parameters, including fasting plasma glucose, glycated haemoglobin A1c (HbA1c), lipoprotein profiles, folate and vitamin B12 levels. Participants who agreed were also asked to complete a short-form FFQ.

During the second study visit, Computer Assisted Personal Interviewing (CAPI) was used to administer a more detailed GHQ which covered various aspects of health including self-perceived health status, adherence to antibiotic and general medication therapy use, morbidity, weight management as well as mental health and wellbeing. Following the CAPI, a physical examination was undertaken with measurements of weight, height, mid-arm and waist circumference, body composition (derived from bioelectrical impedance), grip strength, gait speed and resting blood pressure. A subgroup of participants consented to wear a 24-hour ambulatory blood pressure monitor, which is believed to be less prone to variation compared to a single visit in-office blood pressure measurement. To assess physical activity, the International Physical Activity Questionnaire (IPAQ) was administered to all participants ^13^. In addition, approximately one-quarter of the rescreen cohort accepted to wear accelerometers for one week. Participants were fitted with devices before they left the study centre (i.e., a non-dominant wrist for the GENEActiv accelerometer, the anterior aspect of the midline of the right thigh, halfway between the knee and the hip for the ActivPAL3 activity monitor and the right iliac crest for the ActiGraph accelerometer) and provided with instructions on how to appropriately wear the accelerometers. A comparison of variables collected at baseline and rescreen is shown in Table 2.

**Table 2.** Variables collected at baseline and rescreen.

Variable	Baseline	Rescreen
*Demographics*	✓	✓
*Present circumstance*		
Personal transport	✗	✓
Neighbourhood characteristics	✓	✓
Social support	✗	✓
*Work life*		
Work status	✗	✓
Retirement	✗	✓
Income and pensions	✗	✓
Working environment	✗	✓
Work satisfaction	✗	✓
*Personal health behaviour*		
Smoking (status, type, frequency)	✓	✓
Alcohol (status, type, frequency)	✓	✓
Physical activity levels and duration (IPAQ)	✓	✓
Activities of daily living	✓	✓
*Personal health and medication history*		
Self-rated health	✓	✓
Sickness and disability	✓	✓
Medications	✓	✓
*Chronic conditions*		
Dental health	✗	✓
Falls history	✗	✓
Screening service history	✗	✓
Antibiotic drug use	✗	✓
Anti-inflammatory drug use	✗	✓
Prescription compliance	✗	✓
Multi-vitamins and minerals	✓	✓
Medical history	✓	✓
Self-reported weight management and weight change	✗	✓
Mental health and well-being	✓	✓
Cognitive function	✗	✓
Sleep quality	✗	✓
*Food life*		
Frequency and consumption of 10 food groups	✓	✓
Milk consumption	✓	✓
Fried food consumption	✓	✓
Salt consumption	✓	✓
Location of meal consumption	✓	✓
Specific diet type (vegetarian, vegan, gluten free)	✗	✓
*Physical and other measurements*		
Height (cm)	✓	✓
Weight (kg)	✓	✓
Body composition derived from bioelectrical impedance	✗	✓
Heart rate over 30 secs	✓	✓
Blood pressure (mm Hg)	✓	✓
Waist circumference (cm)	✓	✓
Hip circumference (cm)	✓	✓
Pelvic width (cm)	✓	✓
Calf circumference (cm)	✗	✓
Electrocardiogram	✓	✗
GeneActive accelerometer (Unilever Discover) tri-axial	✓	✓
ActivPAL/ActiGraph accelerometers	✗	✓
Gait speed	✗	✓
Hand grip strength	✗	✓
Ambulatory blood pressure	✓	✓
*Blood, urine and stool*		
Fasting plasma glucose, iron, gamma-glutamyl transferase, creatinine kinase, liver, renal, lipoprotein and bone profiles, magnesium and estimated glomerular filtration rate	✓	✓
Full blood count	✓	✗
HbA1c, vitamin B12, folate and ferritin	✓	✓
Urinary microalbumin, creatinine, microalbumin /creatinine ratio, sodium and potassium	✓	✓
Inflammatory biomarker profiling, including measurement of c-reactive protein, complement component 3, tumour necrosis factor alpha, interleukin 6, adiponectin, leptin, resistin and plasminogen activator inhibitor-1 concentrations	✓	✗
Lipoprotein particle subclass size and concentrations determined using nuclear magnetic resonance spectroscopy	✓	✗
Stool analysis	✗	✓
*Other tests*		
Barthel Index of Activities of Daily Living	✗	✓
Cognitive assessment: Folstein Mini Mental State Exam (MMSE)	✗	✓

## What has it found? Key findings and publications

The clinical data collected in the annual sweeps provided an opportunity to assess general prescribing guidelines according to Screening Tool of Older Persons’ Prescriptions (STOPP) and Screening Tool to Alert to Right Treatment (START) criteria ^14, 15^. Analysis of prescribing data over time demonstrated a high prevalence of potentially inappropriate prescribing among older-aged people in primary care which increased as they progressed to more advanced old age. The findings suggest that routine application of prescribing guidelines in this population has the potential to improve medication appropriateness. The availability of office blood pressure measurements from electronic health records, along with the clinic study visit and ambulatory blood pressure recordings on nearly 1000 individuals, allowed an assessment of hypertension detection, awareness and control and an assessment of the role of ambulatory blood pressure monitoring ^16^. The prevalence of hypertension was high, ranging from 50% to 64% depending on the measurement method and blood pressure thresholds used. The awareness rate varied from 57% to 71%, while 57% to 73% were treated and 46% to 68% were controlled.

The rescreen has also provided new insights into physical activity patterns and cardiovascular health. A recent study analysed sitting, standing and stepping bout relationships with cardiometabolic health markers in older adults ^17^. It showed that sitting (≥10, ≥30 and ≥60 minutes) and standing (≥10 and ≥30 minutes) bouts were detrimentally associated with fasting glucose levels, lipid markers and body composition, with the effects being larger for time spent in ≥60 minutes sitting and ≥30 minutes standing in comparison to shorter bouts. Shorter sitting and standing bouts might be a useful strategy to improve cardiometabolic health in older adults. Another study examined objectively measured time spent in sleep, sedentary time, standing time, light-intensity physical activity (LIPA) and moderate-to-vigorous intensity physical activity associations with markers of cardiometabolic health using compositional analysis of data from the Mitchelstown cohort ^18^. It found that BMI, body mass and fat mass were negatively associated with LIPA while being positively associated with standing time. These findings are supportive of engagement in LIPA to improve body composition in older adults.

To date, two publications have focused on the gut microbiota composition utilising data from participants who provided stool samples at rescreen ^19, 20^. The results of the first study, which investigated gut microbiota associations with metabolic syndrome and obesity, indicate greater microbiome diversity in metabolically healthy non-obese individuals relative to their unhealthy counterparts ^19^. The second study explored relationships between objectively assessed physical activity behaviours, as measured by the activPAL3 activity monitor over 7-days, and the gut microbiome ^20^. Overall, findings from this study suggest that increased levels of LIPA are able to improve specific gut microbiota with beneficial properties on health in older adults ^20^. 

Data from the rescreen have also been used to assess established and novel measurement methods, including a shortened FFQ and BMI self-assessment ^21^. A recent study also compared the prevalence and correlates of prediabetes and diabetes using different diagnostic methods and found discordance between prediabetes prevalence estimates with respect to cut-offs recommended for HbA1c and fasting plasma glucose by the American Diabetes Association and World Health Organization International Expert Committee ^22^.

## What are the main strengths and weaknesses?

There are two major strengths to the current study. First, as with the baseline sample, the rescreen study is embedded in a single primary care centre. This enables passive follow-up of study participants through the use of electronic health records. In addition, the use of electronic records provides details of ongoing changes in medication prescribing and specialist referral. Second, several new measures were added to the rescreen study. These new measures include cognitive function decline, medication prescribing and adherence and collection of stool samples for gut microbiome analyses. These data provide opportunities to address a range of research questions related to population ageing.

The main weaknesses are the small study population size and its regional character. Despite being one of the largest prospective cohorts in an Irish middle-aged to elderly population, the Mitchelstown cohort remains a relatively small study on an international scale. In addition, as data were collected from a single primary care centre in one region, they may not be nationally representative. However, Ireland represents a generally ethnically homogeneous population ^23^. In addition, previous research suggests that approximately 98% of Irish adults are registered with a GP and that, even in the absence of a universal patient registration system, it is possible to perform population-based epidemiological studies that are representative using our methods ^24^.

## Ethics policies

Ethics committee approval conforming to the Declaration of Helsinki was obtained from the Clinical Research Ethics Committee of University College Cork. The Cork and Kerry Diabetes and Heart Disease Study is General Data Protection Regulation compliant.

## Consent

A letter signed by the contact GP in the clinic was sent out to all selected participants with a reply slip indicating acceptance or refusal. All participants gave signed informed consent, including permission to use their data for research purposes.

## Data Availability

All data collected at baseline and rescreen are maintained and stored at the School of Public Health, University College Cork. Due to small numbers with regard to certain variables collected, the datasets are not shared publicly in order to ensure study participant anonymity. However, a list of variables and coding manuals are available upon request. The Cork and Kerry Diabetes and Heart Disease Study has several established collaborations. For new collaborations and information on data sharing please contact the data manager at: s.millar@ucc.ie.
