# Supplementary material for: Cohort profile update: The Cork and Kerry Diabetes and Heart Disease Study
Source: HRB Open Res. 2023 Apr 21;6:25. [Version 1] doi: 10.12688/hrbopenres.13626.1 (PMC10435920; doi:10.12688/hrbopenres.13626.1)
Supplement: Peer review history

# Peer review: Reviewer response for version 1

**DOI:** 10.21956/hrbopenres.14898.r35491

**Published:** 2023-08-21

**Authors:** Elijah N Ogola

**Affiliations:** 1 University of Nairobi, Nairobi, Kenya

Thanks for the opportunity to review this manuscript. I have a few specific comments to make.

- The current report is based on the Mitchelstown cohort rather than the original Kerry and Cork cohort. This is not clearly explained.
- I wonder about the accuracy of accelerometers in assessing usual physical activity. Doesn’t knowing that one is being monitored introduce bias? This point should be discussed.
- The assessment of adherence is not described.
- One of the difficulties I had, is sort of inherent in the nature of the publication. It is a summary of a complex project. The details, both in terms of methodology and results are therefore brief. It is therefore difficult to adequately critique these.

It is otherwise an interesting and important project.

Are sufficient details of methods and materials provided to allow replication by others?

Partly

Is the rationale for creating the dataset(s) clearly described?

Yes

Are the datasets clearly presented in a useable and accessible format?

Yes

Are the protocols appropriate and is the work technically sound?

Yes

Reviewer Expertise:

Cardiovascular risk factors. Clinical research in heart failure and hypertension.

I confirm that I have read this submission and believe that I have an appropriate level of expertise to confirm that it is of an acceptable scientific standard.

# Peer review: Reviewer response for version 1

**DOI:** 10.21956/hrbopenres.14898.r34837

**Published:** 2023-08-17

**Authors:** Evangelia Kalaitzoglou

**Affiliations:** 1 University of Kentucky, Lexington, USA

The update on the Cork and Kerry Diabetes and Heart Disease Study is an important addition to the existing information about these participants offering a long-term follow up on certain variables related to cardiovascular health, but more importantly, reports on several new variables related to general health, with information including, but not limited to, cognitive function, medication and dietary variables, physical activity and fitness variables, work life variables and general health conditions.

 There are important findings that arise from the re-screen of the cohort regarding medication prescription for older individuals, blood pressure assessment and hypertension diagnosis and management trends, physical activity and cardiometabolic health and associations between gut microbiome and physical activity or obesity.

 Very minor additions would improve the quality of this report. Adding the ethnicity and race for the participants If available in Table 1, in addition to other demographics such as age and sex that are already reported would be very informative. The authors should also elaborate on the reasons for the microbiome study excluding participants that were younger than 55 years of age.

 Lastly, the authors might want to rephrase their conclusion section in the abstract and remove this phrase from the conclusion and add to the methods:  “All data collected at baseline and rescreen are maintained and stored at the School of Public Health, University College Cork”.

Are sufficient details of methods and materials provided to allow replication by others?

Yes

Is the rationale for creating the dataset(s) clearly described?

Yes

Are the datasets clearly presented in a useable and accessible format?

Yes

Are the protocols appropriate and is the work technically sound?

Yes

Reviewer Expertise:

Pediatric Endocrinology and Diabetes

I confirm that I have read this submission and believe that I have an appropriate level of expertise to confirm that it is of an acceptable scientific standard.

# Peer review: Reviewer response for version 1

**DOI:** 10.21956/hrbopenres.14898.r35489

**Published:** 2023-08-17

**Authors:** Jean Jacques Noubiap

**Affiliations:** 1 Department of Medicine, University of California San Francisco, San Francisco, California, USA

This paper presents an update of the Mitchelstown cohort which is part of the Cork and Kerry Diabetes and Heart Disease Study. The authors present the reasons for change in data collection, the new areas of research and new data collected, participants' characteristics and the main findings as well as publications from the updated cohort. This is a well-written cohort profile article which is worth of indexing.

 I have no concerns.

Are sufficient details of methods and materials provided to allow replication by others?

Yes

Is the rationale for creating the dataset(s) clearly described?

Yes

Are the datasets clearly presented in a useable and accessible format?

Yes

Are the protocols appropriate and is the work technically sound?

Yes

Reviewer Expertise:

Hypertension, diabetes, heart failure, atrial fibrillation, cardiovascular disease

I confirm that I have read this submission and believe that I have an appropriate level of expertise to confirm that it is of an acceptable scientific standard.
